# Improving hospital-based care for patients with injection-drug related infections: provider perspectives

**DOI:** 10.1186/s13722-026-00651-9

**Published:** 2026-02-09

**Authors:** Giselle Appel, Kayla Hutchings, Cristina Chin, Alexis Vien, Matthew Scherer, Jonathan Avery, Shashi N. Kapadia

**Affiliations:** 1 Northwell, New Hyde Park, NY USA; 2 Weill Cornell Medical College, New York, NY USA; 3 University of North Carolina School of Medicine, Chapel Hill, NC USA; 4 Columbia University Medical Center, New York, NY USA

**Keywords:** Substance use disorders, Injection drug use, Addiction, Severe bacterial infections, Stakeholders, Clinical hospital programming

## Abstract

**Background:**

Injection-related severe bacterial infections (SBIs), including skin and soft tissue infections, endocarditis, and osteomyelitis, are rising in prevalence in the United States and disproportionately affect people who inject drugs (PWID). Hospitalization for SBI presents a critical opportunity to engage patients in addiction and infectious disease care, yet healthcare systems often fail to capitalize on this moment.

**Methods:**

This qualitative study explores the perspectives of 22 clinical stakeholders, including physicians, surgeons, nurses, and social workers, on barriers and strategies to improve care for hospitalized people who inject drugs (PWID) with substance use disorders (SUDs). Participants were recruited through purposive sampling from a large urban healthcare institution and selected external sites. Semi-structured interviews were conducted and analyzed using a thematic analysis approach.

**Results:**

Three core themes emerged: empowering clinical teams, enhancing patient care, and improving interdisciplinary workflows. Participants called for stronger provider education on substance use disorders, stigma reduction, and communication; expanded access to addiction consults, harm reduction tools, peer navigators, and flexible discharge options such as OPAT with coordinated follow-up. Barriers included limited staffing, inconsistent access to addiction specialists and peers, and institutional inertia around discharge and reimbursement. Stakeholders highlighted telemedicine and structured interdisciplinary communication as means to prevent care fragmentation, noting that patient-directed discharges often stem from unmanaged withdrawal, stigma, and mistrust within existing hospital systems.

**Conclusions:**

Our findings align with prior research on addiction consult models and highlight emerging support for hybrid care delivery models that integrate harm reduction, peer support, and telehealth. However, practical and structural limitations, such as policy constraints, funding gaps, and misaligned institutional incentives, remain significant obstacles. This study contributes to a growing body of literature calling for targeted, feasible interventions that can transform hospital-based care for PWID with SBIs. Future work should address patient perspectives and evaluate outcomes of proposed strategies to guide institutional investment and policy reform.

**Supplementary Information:**

The online version contains supplementary material available at 10.1186/s13722-026-00651-9.

## Background

Injection-related severe bacterial infections (SBIs) are among the most serious consequences of the injection drug use epidemic and are increasing in incidence [1–4]. Approximately 1.5% of the United States population (or 3.7 million people) use drugs by injection, and such individuals have a disproportionate burden of infectious diseases on an individual and health systems level [5]. SBIs include skin and soft tissue infections (SSTIs) requiring medical or surgical intervention (such as cellulitis, abscess, or necrotizing fasciitis), bacteremia, endocarditis, or bone/joint infections (such as osteomyelitis or septic arthritis), with the highest prevalence being that of SSTIs [6, 7]. Hospitalization represents a unique capture point/moment to initiate addiction interventions, yet most healthcare systems fail to maximize the potential of this opportunity.

The incidence of hospitalizations for SSTIs in those who inject drugs has been steadily rising across the United States over the past decade. For example, from 2015 to 2020, hospitalizations in Philadelphia for those with SSTIs, osteomyelitis, bacteremia and sepsis, and infective endocarditis among people who use drugs increased by 91%, 73%, 253%, and 240%, respectively [8]. By 2030, endocarditis associated with injection drug use is predicted to cause roughly 250,000 deaths in the United States [9, 10]. Reasons for elevated risk for SBIs among patients who inject drugs are multifactorial, including repetitive tissue damage, non-sterile drug diluting water, lack of skin hygiene, and re-use of injection equipment, all of which lead to increased susceptibility to bacterial contamination [11].

While direct inoculation leads to increased rates of incidence of SBIs, delaying care exacerbates poor outcomes for patients. There are many reasons why patients may avoid seeking out care, such as lack of access to care, unstable living environments, and limited knowledge about the connection between bacterial inoculation from injection drug use and resulting serious infections [5]. Another core reason for delaying care is the pervasive stigma that exists within the medical community toward these patients. Individuals with substance use disorders (SUDs) are rated lower than other medical and psychiatric disorders by physicians on the Medical Condition Regard Scale, reflecting the stigma experienced by this population [12]. Those from marginalized populations may also face compounded discrimination [13–15] and, as a result, mistrust of the hospital system often develops. Mistrust in clinical decisions, compounded by potential mismanagement of cravings and withdrawals, frequently leads patients to self-direct their discharge without the necessary safeguards in place, and therefore, higher rates of readmission [16].

Systemic healthcare inequities, such as the limited availability of outpatient parenteral antibiotic therapy for persons who inject drugs (PWID) with SBIs, further compound poor outcomes [17]. Parenteral antibiotic therapy via a peripheral intravenous central catheter (PICC) line is a cost-effective solution associated with shorter hospital stays and decreased readmission rates [18]. Due to concerns about potential misuse or complications, clinicians are often hesitant to discharge patients home with PICC lines, which can result in prolonged hospitalizations [19]. Compounding this issue, PWID often receive suboptimal delivery of addiction-related care, particularly medications for opioid use disorder, despite their role in reducing morbidity and mortality in the peri-hospitalization period [10, 20, 21]. Together, long lengths of stay with insufficient addiction treatment lead to the risk of increased cravings, withdrawal discomfort, and patient-directed discharge. In recent years, peer support specialists (i.e., individuals with lived experience of substance use disorder and recovery) have been gaining popularity as a key contributor to hospital-based addiction support [22, 23]. These individuals are often integrated into the discharge planning process, offering support and building trust with the patient to help bridge the gap between inpatient care and outpatient follow-up. However, despite their impact, the integration of these advocates is inconsistent and they’re often underutilized [23].

While several pilot studies have explored interventions that may improve care, there is limited data on the needs and barriers of healthcare stakeholders in implementing novel care models for this population. Providers are responsible for implementing infection and addiction care; therefore, their insights are invaluable in finding practical strategies for improved care. This may encourage clinical leadership to take practical steps to address specific needs forthis unique population. This study aims to describe strategies that address gaps in care and the barriers limiting them.

## Methods

This study used a qualitative research design to carry out semi-structured interviews with providers involved in the treatment of severe bacterial infections for hospitalized persons with injection drug use. Interviews were conducted using a detailed interview guide that focused on barriers and strategies to improve care for hospitalized people who inject drugs (PWID) with substance use disorders (SUDs) (Appendix A). The study investigators, all physicians who work clinically with infectious disease patients, (S.K., M.S., A.V.) developed the interview protocol. The Institutional Review Board approved the study protocol at the Biomedical Research Association of New York (BRANY, #22-08-093-380).

### Population and sampling strategy

Participants included clinical leadership and healthcare workers, specifically hospitalist providers, addiction medicine providers, nursing leadership, and social work leadership, primarily from an urban, single, multi-campus, academically affiliated hospital system in New York City (*n* = 18). The participants work at any of two hospital campuses that contain 2056 inpatient beds and have closely affiliated outpatient services. Individuals were identified based on the investigators’ contacts and suggestions from clinical leadership. We also interviewed clinical providers at 4 external institutions who were established thought leaders to supplement our data with an extra-institutional perspective (*n* = 4). These institutions were selected based on a literature review at the time of the study, which identified specific published interventions that focused on care for PWID with SBIs – all were U.S. academic hospitals, but the specific interventions are not mentioned to maintain participant confidentiality.

### Data collection

Participants were recruited using purposive sampling through email and provided informed consent. Participants were compensated $50 for their participation. Each interview lasted 30–45 min and was conducted virtually using Zoom software with authors (G.A. and K.H.) trained in qualitative interviewing. The interview protocol was piloted with two infectious disease physicians to optimize interview questions. Pilot interview data were included in the analysis.

### Analysis

Interviews were recorded and transcripts were de-identified. Each transcript was then transcribed using a professional transcription software called Ubiqus Translation Services, imported into NVivo 12 Pro software, and read line by line by investigators within a week of the interview’s completion to ensure accuracy. The transcripts were coded using thematic analysis in NVivo (G.A. and K.H.). These coders met to review and resolve any discrepancies, promoting the internal validity of the data interpretation. A secondary coding team (S.K, C.C, G.A, K.H, A.V, M.S) reviewed emerging codes to provide big-picture feedback and resolve coding discrepancies. Coding discrepancies were resolved through ongoing meetings where the discrepancies were reviewed and resolved through group consensus until a high interrater reliability score was achieved (measured using the kappa coefficient of 0.80 or above). Codes were grouped into categories, and emergent themes were identified through bi-weekly team meetings and consensus-based approach. The study size was informed by information power, which is reflected in the specificity of our sample population and the quality, as well as the length, of the dialogue between the interviewer and participant [24, 25].

## Results

A total of twenty-two participants completed the interview. All 22 worked in clinical roles, with 7 holding clinical leadership positions. (Table 1). The majority of participants were hospitalist physicians (*n* = 8), infectious disease physicians (*n* = 6), or psychiatrists (*n* = 4). Surgeons, patient care directors, and social workers were also represented. Participants were split roughly evenly between the two hospital sites (*n* = 8 and *n* = 10). The four external participants held leadership positions related to SBI care at their institutions. The average years of experience after training for each participant was 7 years, meaning most are mid-career professionals.

**Table 1:** Participant professional role and affiliations

	Hospital Site 1	Hospital Site 2	External
**Category**			
Clinical Leadership	3	0	4
Other Clinician	5	10	0
**Total**			22
**Specialty**			
Hospitalist	3	5	0
Infectious Disease	2	1	3*
Cardiothoracic Surgery	1	1	0
Addiction Psychiatry / Addiction Medicine	1	1	2*
Medical Intensive Care Unit	1	0	0
Patient Care Director	0	1	0
Social Worker	0	1	0
**Total**			22

*Note: One interviewee from the ‘External’ site specializes in both Addiction Medicine and Infectious Disease. The totals in this chart reflect the number of interviewees

Participants shared insights into strategies for enhancing care for this patient population within the hospital setting. Analysis of the interviews revealed themes broken down into three overarching categories: “Empowerment of Clinical Teams,” “Direct Patient Care,” and “Improving Clinical Workflow for Interdisciplinary Teams”.

### Empowering clinical teams to take care of patients

#### Education on opioid use disorder and medication-assisted treatments

One of the prominent barriers across interviews highlighted the limited availability of clinical providers who were trained in treating comorbid substance use and mental illness. Many clinicians emphasized the need for greater education on opioid use disorder and medication-assisted therapies, citing that prior medical education was not sufficient in covering the basics of addiction medicine. Further, one participant noted the benefit of “*shadowing folks [patients] who were going to medical withdrawal facilities… and attending groups with patients”* during residency. *- Infectious Disease Physician.*

One participant cited clinical data that “just by educating providers over one hour, you can reduce stigma and increase their comfort in treating patients who inject drugs” - *Infectious Disease Physician*,* Associate Professor in Medicine*,* in the Division of Infectious Diseases (External).* Another saw a need for continued education on “communication with patients…that may be withdrawing, or may be deemed difficult, how to gain their trust and help them ultimately achieve better outcomes” - *Physician Assistant on the Infectious Disease Consult Service.*

#### Improving bedside relations between patients and the primary team

Beyond improving knowledge, some providers also hoped that educational interventions could help mitigate the stigma that patients experience, while enabling providers to relate to patients more effectively. One participant cited a barrier to offering services that address this care need, stating, “*Some hospitals don’t want to have an active*,* robust addiction consult service because they think it might attract more of these patients who*,* again*,* potentially may be cost-losing*,* if they are admitted to the hospital.” - Physician Hospitalist.*

Many participants mentioned the potential utility of peers in helping patients navigate their path to rehabilitation while they are under the care of the primary team. Peers are defined as individuals in various stages of substance use recovery with experiential knowledge about life with an addiction. Thus, these individuals offer a unique perspective that is not readily available to hospital staff.

*I would love to see an inpatient peer navigator for patients.​ We have peer folks who go to the emergency department. But I think having a peer at the hospital would be super helpful. I know that there are programs that have made this happen*,* and it’s been something we’re advocating for. But we’ve been told because of reimbursement and financial issues*,* it has not yet been possible. But what would be on my wish list is to have a peer. - Infectious Disease and Addiction Medicine Physician (External).*

Even when there are peer services available, participants expressed the need for easier accessibility, noting peers aren’t used often, *“Because it takes extra work. For the provider*,* you have to fill out a form*,* and if that were easier*,* I think that would be really nice. - Consultation Liaison and Addiction Psychiatrist.*

### Direct patient-care interventions

#### Earlier and more available addiction medicine consults to address effective management of withdrawal while in the hospital

While increased education on opioid use disorder management was echoed across interviews, participants also recognized the need for earlier consults with addiction medicine specialists. Behavioral issues, particularly those perceived by healthcare staff as verbally abusive, were identified as a sequela of addiction and thus thought to be better managed by “getting psychiatry consultants on board earlier during their stay, just to provide recommendations on how to deal with this type of behavior.*” - Registered Nurse and Patient Care Director.*

*Because we are the ones who are receiving workspace or verbal abuse*,* or even sometimes physical abuse*,* too. We are just trying to make sure that they’re safe*,* while we are suffering abuse for the patient’s health. You know*,* being able to leave the floor*,* being able to leave the unit. And I hate to get security involved*,* but it ends up being that way sometimes. - Registered Nurse and Patient Care Director.*

The above quote refers to challenges with patients who use drugs requesting to leave the unit (potentially to use drugs), which may be governed by specific policy or institutional practice, and which can create conflicts between the patient and the care teams. Provider participants recognized that patients with substance use are often inclined to self-direct their discharge when cravings or discomfort with withdrawal are not sufficiently addressed. Further, even when addiction consults are made, participants recognize that inadequate staffing on this service and limited time during consults contribute to this problem.

*I think one thing we could use is other clinicians*,* not just physicians*,* to collaboratively engage them in the care of the patients and support their time to counsel them. I think it’s great to support MDs*,* but it doesn’t have to be all MDs doing that. I think that was very important*,* and then I think having outpatient resources*,* and having it be holistic. - Physician Hospitalist.*

Although many hospital providers felt that increased availability of addiction consultation could be beneficial, addiction specialists themselves emphasized the value of being more directly involved in each patient’s care. One consultation liaison and addiction psychiatrist described making a point to see hospitalized patients multiple times per day for supportive psychotherapy. One provider, who was a psychiatrist, emphasized the benefits of actively making time to provide supportive psychotherapy for hospitalized patients:

*I see [the patients] between twice or three times a day for supportive psychotherapy because it’s hard to be in the hospital for such a long time. So not only to remind them that it’s important to complete the treatment*,* but also to troubleshoot with them*,* using cognitive behavioral therapy strategies to cope with prolonged hospitalization and some minor stressors*,* like problems with nurses*,* problems with the primary team. - Consultation Liaison and Addiction Psychiatrist.*

*For psychotherapy to be effective*,* the patient needs to be comfortable. So I usually start as soon as the withdrawal symptoms are under control…So it may take sometimes two*,* three days*,* four days – it’s not immediate. It’s a mix of supportive*,* motivational*,* and cognitive behavioral therapy. - Consultation Liaison and Addiction Psychiatrist.*

#### Offering harm-reduction tools and education as preventative resources

Participants cited the disadvantages of poor social support and environmental triggers that complicate patient care after discharge. While clinical providers may have limited ability to intervene in the structural and social conditions that patients return to upon discharge, they emphasized the importance of offering harm-reduction tools to reduce the risk of overdose or re-infection.

*We also have a Narcan program here*,* an opioid overdose prevention program. So*,* we also provide education to all these patients*,* and we provide at least one Narcan kit upon discharge*,* even if/when they leave against medical advice. So*,* this can potentially prevent overdoses. - Consultation Liaison and Addiction Psychiatrist.*

As stated in the above quote, suggestions include the provision of naloxone or fentanyl testing strips to decrease the risk of opioid overdose, and information on nearby harm-reduction sites/programs.

Participants also emphasized the importance of initiating conversations for medication for opioid use disorder (e.g., a rapid methadone initiation or low-dose buprenorphine induction), to prevent ongoing intravenous drug use.

#### OPAT and PICC line decision-making

Participants described outpatient parenteral antibiotic therapy (OPAT) delivered via peripherally inserted central catheter (PICC) line as a particularly challenging practice to implement in this population. Some clinicians emphasized the dilemma of balancing infection treatment needs, with perceived and real risks of PICC line misuse. Some stated they were willing to discharge patients with a PICC line if they were on methadone or had a remote history of IVDU.

*If they’re on methadone – if they’re not using injection drugs – then yeah*,* we would kind of give them the benefit of the doubt. And those people*,* I would send home with the PICC – Cardiothoracic Surgeon.*

Others described using structured tools and collaboration with addiction colleagues to guide the decision whether or not to discharge a patient with a PICC line. Some acknowledged that the fear of patients injecting into their lines was often not grounded in objective evidence. One said, *“There is no research to suggest they’re any more high risk than anyone else… none of my patients actually eloped with a PICC line and disclosed that they used through it” – Social Worker.*

Despite these nuances of inserting the PICC line, there were other barriers to consider. Infusion companies, nursing facilities, and some hospitals often refused to accept patients with ongoing or recent substance use, leading to prolonged admissions. As one provider explained, “*Historically*,* it was putting in a PICC line and then deciding where they would receive their long-term antibiotics… recognizing that 99 out of 100 times they can’t go home because they’re active IVDU” – Physician Hospitalist.* Another stated, “*The patient case has to be accepted by the infusion company… and homeless patients often cannot have infusions arranged in shelters” -Physician Hospitalist*, sometimes leaving providers with no safe discharge pathway. In response to the above dilemma, some participants reported turning to long-acting injectable antibiotics, like dalbavancin, despite inconsistent insurance coverage. One clinician said, “*It is often worth it for the hospital to eat the cost of the dalbavancin*,* rather than eat the cost of a longer hospital admission” -Infectious Disease Physician.*

In addition, one provider was amenable to sending patients home with PICC lines, despite having a history of IV drug use.

*If they’re on methadone and if they’re not currently using IV drugs*,* then yeah*,* we would give them the benefit of the doubt. And those people*,* I would send home with the PICC*,* if they have not had recent use. We rely on the patient if they’re taking [their MOUD] regularly. I found that most of them are pretty honest with whether they’re actively using. So*,* that’s how we’ve been doing it. - Cardiothoracic Surgeon.*

Across interviews, OPAT and PICC line management were framed less as a harm reduction strategy and more as a continuity-of-care issue for this patient population. This was an issue shaped just as much by institutional policies and stigma as by the evaluation of patient reliability in using their line appropriately.

#### Addressing structural barriers to post-discharge engagement

A lack of reliable phone services, limited funds to attend such appointments, and housing insecurity prevent many patients from accessing follow-up services. As one hospice physician explained, hospitals must take initiative in “making sure the patients have whatever antibiotics or medications they need on discharge…and transportation to their first appointment with their infectious disease or primary care doc.” The same physician noted that models involving long-acting injectable antibiotics, paired with transportation infrastructure, could ensure follow-up.

*I think if there were a clinic where you could go and get the injectable IV antibiotic if it was appropriate*,* that would last a week*,* two weeks*,* whatever. But we had a way to transport them back and forth from wherever to the appointment to ensure that they got the appropriate treatment and appropriate follow-up. I think that would be huge. - Hospice Physician.*

These reflections point to how socioeconomic disadvantages obstruct continuity of care, and point to transportation as an actionable target for health systems seeking to reduce readmissions.

#### Leveraging telemedicine services to improve access to care

Participants described how telemedicine could be used to support patients during in-hospital services and, in some cases, to services after discharge. Virtual platforms were seen as a means to provide timely consultations, triage specific needs, and extend the reach of addiction specialists.

*I think telemedicine is a great way that can be facilitated more equitably across our campuses. In a dream world*,* in-person and virtual support; in a second version*,* at least virtual support across campuses*,* for sure. I think they need to protect people’s time and pay them because I think right now we’re just kind of informally curb-consulting people*,* and that’s not sustainable for their careers. So*,* I think that’s one piece*,* and it’s better for patient care for this to be documented fully in the chart and done the right way. - Physician Hospitalist.*

Some envisioned a model of clinical care where a single provider could offer continuity of care, beginning while in the hospital stay and continuing in their post-discharge course. This could serve as a bridge to outpatient services, while also functioning as a model for sustained relationships with a single provider.

*To me*,* that’s the direction of the future – there is a virtual hospitalist that sees the patient when they’re in the hospital*,* and they see them later*,* and they have that person’s number to call them at any time*,* or we give them a bracelet with that number. What can we do to plug patients in so they have someone*,* they have a lifeline*,* and they develop care and trust with that person? I think we need something like that. - Physician Hospitalist.*

Others highlighted that telemedicine services could provide care beyond appointments, but rather a possible multidisciplinary hub for coordination of patient care and collaboration across providers within behavioral health and medicine.

*Not sure whether I can name one*,* but having an addiction medicine consult*,* who then coordinates outpatient addiction care*,* is probably the most important intervention because usually*,* those programs have collaborations with social work and psychiatry to help address the combination of problems that these patients face. That would be the biggest intervention that could be beneficial to these patients.” - Hospitalist Physician.*

### Improving clinical workflow for interdisciplinary teams

#### Improving communication among staff through frequent interdisciplinary meetings to achieve shared understandings of and improved efficiency in carrying out patient outpatient disposition plans

Arranging discharge to facilities that accept patients who are on methadone therapy is complex, as many facilities hold specific policies regarding what type of patient they are willing to accept. Participants expressed the need for interdisciplinary meetings to “understand where things fall through the cracks, where we can work together as partners more effectively to provide the same care that others deserve” *- Infectious Disease Physician.* The multidisciplinary team could “address the problem, probably obstacles, and create a contingency plan in the event of AMA or if the patient refuses surgery” to ensure the providers are all on the same page. - *Infectious Disease Physician.*

One participant suggested that if each specialist on the treating team could make outpatient referrals, instead of going through the primary team, it would provide more effective care.

*There might be a way to*,* as I said*,* remove that you have to go through the primary team to make that referral*,* if it’s understood that this is never really detrimental and always positive*,* if you can*,* then just—that would be a way to help with that. - Infectious Disease Physician.*

#### Establishing standardized protocols for follow-up with patients who leave against medical advice

Many patients with substance use disorders will opt for self-directed discharge, which may not leave enough time for the clinical team to coordinate with pharmacies or outpatient services. One system-wide failure cited by a participant is the lack of availability of follow-up appointments in the resident-run clinics. “*Attendings face difficulty arranging appointments before a patient’s discharge*, and therefore *expressed sympathy for how complex the process is for residents*.” *- Cardiothoracic Surgeon.*

One participant expressed the need for aftercare or follow-up that is integrated into the health care system, stating, “*I would love to have a nurse case management model that follows patients and ensures that they don’t fall off the rails. Because I think that’s happening a lot. And I think there are times that we could pull people back in*,* but if we don’t have that continued relationship with them*,* it can be challenging.” - Addiction Medicine Physician.*

Although patients may self-direct their discharge, there is the issue of how they will manage their infection without parenteral antibiotics in the outpatient setting.

*Usually*,* we have the Infectious Disease team on board*,* especially if we’re giving Dalvance (dalbavancin). But if we do end up doing that*,* we often have the ability to have a follow-up appointment in our ID clinic. So*,* if they leave*,* if they want to leave*,* we’ll still try to either prescribe them – give them the IV antibiotic and try to do an oral antibiotic*,* and then also give them follow-up with our fellows or attendings in the ID clinic. - Hospitalist Physician.*

#### Coordinating outpatient substance use treatment by addiction teams

For those at risk for substance relapse or continued use, coordination of outpatient services with addiction specialists is needed. One participant emphasized the importance of longitudinal relationships with patients in the outpatient setting, noting that trust-based care is essential for positive outcomes.

*Longitudinal trusting relationships are probably what’s most valuable to helping those patients. I think videos*,* paper*,* and verbal talks by others are ineffective. - Hospitalist Physician.*

*As far as services*,* the big thing would be transitions of care… What can we offer these patients when they leave the hospital…it’s like we have nothing to offer unless they can pay cash for some fancy rehab. Our social worker can provide them with the names and numbers of places. But it does always feel very like*,* “Here is a phone number*,* good luck”. - Hospitalist Physician.*

*So*,* some like a system or care manager*,* or something we could offer these patients when they leave the hospital to help them stay healthy and stay out of the hospital would be really helpful*,* I think.”​​ - Intensivist Physician.*

The above participants expressed skepticism about one-time interventions, suggesting that brief educational materials and even names/numbers of locations are ineffective without the foundation of a sustained therapeutic relationship. Whether an individual’s first or fifth hospitalization for SBI is related to their injection drug use, hospital admission could serve as an opportunity to reconnect with longitudinal addiction treatment, but our participants largely saw this as an untapped opportunity.

## Discussion

These results offer insights into the multifaceted barriers and promising strategies to improve the care of people who inject drugs (PWID) with severe bacterial infections. Although the majority of our interview questions focused on infection management, the themes that emerged were more focused on direct patient care interventions related to substance use disorders, highlighting the intersectionality of this issue in the hospital setting. These strategies and their relationship to common patient care issues are further summarized in Table 2, along with participant-reported barriers to implementation, and possible solutions to those barriers.

**Table 2:** Proposed strategies and solutions to implementation barriers

Theme	Proposed Strategy	Problems Addressed by the Proposed Strategy	Barriers to Strategy Implementation	Suggested Solutions to Barriers
1.1	Provider education on OUD and MAT	Limited provider knowledge	Provider time and ability to engage in education programs	Brief structured trainings
1.2	Utilization of Peers	Stigma experienced by patients	Challenges in reimbursement for peer roles; administrative burden of referrals	Simplify the referral process Secure institutional funding or contracts with outside organizations
2.1	Earlier addiction medicine consults	Patient withdrawal symptoms	Inadequate staffing and limited time for consults	Leveraging non-physicians such as social workers and case managers
2.2	Offer harm reduction resources and education	Difficulty connecting to services after discharge	Stigma among staff; inconsistent distribution of harm-reduction tools	Increased staff education
2.3	Collaboration with addiction medicine colleagues on PICC line discharge plan	Challenges offering outpatient intravenous antibiotics.	Post-discharge care facilities may refuse to accept discharged patients, disrupting continuity of care	Increased use of long-term injectable antibiotics and partial oral treatment
2.4	Provide more transportation resources	Low access to treatment post-discharge	No existing transport programs; patient financial and housing instability	Develop transportation linked programs (i.e. vouchers or shuttles)
2.5	Utilization of telemedicine	Low access to Increase the likelihood of patient retention in treatment post-discharge	Informal consults instead of structured telemedicine workflow	Formalize telemedicine consult pathways; ensure protected clinical time; create more robust infrastructure
3.1	Hold interdisciplinary meetings to facilitate cross-specialty coordination of care	Frequent disposition challenges, including facility acceptances	Siloed workflows	Implement regular interdisciplinary rounds
3.2	Integration of aftercare preparation into routine workflows	High likelihood of patient directed discharges	Limited time for aftercare preparation	Standardized pre-AMA protocols
3.3	Increased involvement of the hospital care team on transitions to outpatient care	Fragmentation and lack of coordination in service access after discharge	Social workers are often limited to providing “names and numbers,” which patients may not be able to access	Care-manager or nurse-case-manager models; strengthened linkages to outpatient addiction and primary care services

Our findings concerning the perspectives of numerous stakeholders in patient care extend the work of those who have explored healthcare provider perspectives in caring for patients with serious injection drug-related infections. Hayden (2020) [26] and Wurcel (2023) [27] explored and provider decision-making and distress when evaluating their duty to provide clinical care versus a sense of futility in repeat surgeries/care for patients with injection-related infections [26, 27]. Our present study builds upon these works by expanding the lens to encompass the entire hospitalization episode, rather than focusing solely on the specific decisions surrounding cardiothoracic surgery.

One of the major themes in our study addresses direct patient care interventions and skills, particularly addiction medicine or psychiatry consultation, harm reduction tools, and peer support. These findings extend the work of prior research studies that evaluated the impact of addiction consult teams on patient outcomes and retention in SBI care [28–30].

The emergence of “peer support” themes underscores the value of shared, lived experiences offered to patients in real time, which helps bridge distrust between the medical system and patients [29]. Although peer support is generally viewed as an evidence-based complement to recovery, Bao et al. (2024) found that the integration of such resources is limited in the hospital setting due to poor reimbursement challenges (often because peer services are covered by Medicaid if the hospital contracts with outside organizations) and complexities with integrating such individuals into workflow (e.g. administrative issues) [31].

Although consults emerged as a considerable issue for treating this population sufficiently, gaps in staffing and inconsistent availability could undermine their impact. Notably, first-line providers such as hospitalists discussed the importance of increasing capacity, while at least one addiction psychiatrist noted the value of frequent patient contact, suggesting that there may be a volume-quality trade-off for addiction consultation. One way to address this issue is by leveraging non-physicians, such as social workers or case managers, to fill the gap. For example, McNeely et al. (2024) described the CATCH program (a comprehensive team of social workers, peer specialists, and counselors) to manage SUDs in the hospital [30]. This model demonstrated improved initiation and engagement in MOUD, as well as reduced utilization of acute care services. That said, follow-up studies cited difficulties to long-term success, including “limited resources (e.g., medical comorbidities, homelessness), insurance, CATCH team role confusion, and infrastructure deficits (e.g., availability of physical space)”, which points to factors both inside and outside the hospital that need to be considered when developing similar models [32].

Hybrid consultation models, such as those using telemedicine and virtual care coordination, could be integrated to mitigate some of the barriers to long-term follow-up seen in fully in-person models. Virtual follow-up models have shown some success in psychiatry and other fields [33, 34], and evidence on SBI or addiction-related hospitalization is emerging [35]. Recent literature has also studied telemedicine as a means of improving access to treatment for opioid use disorder in populations facing barriers to in-person care. In a study by Hammerslag et al. (2023), telemedicine initiation of buprenorphine treatment for opioid use disorder among Medicaid enrollees during the COVID-19 pandemic was associated with higher odds of treatment retention at 90 days, but was not associated with a change in the odds of opioid-related nonfatal overdose [36]. These findings suggest that telemedicine is a viable strategy for expanding access to buprenorphine treatment, particularly in populations facing barriers to in-person care, without compromising safety in terms of overdose risk.

Aligned with research that has found outpatient parenteral antimicrobial therapy (OPAT) to be acceptable in some cases [37], our participants did not overtly express fears that patients would misuse the IV. Instead, some participants expressed their support for discharge with IVs. This echoes findings from a pilot study by Fanucchi et al. (2019), whose participants completed OPAT while receiving concurrent OUD treatment, suggesting that carefully designed and integrated programs can mitigate the risks that are often (and in our study) cited as barriers [38]. They argued that keeping patients in the hospital to complete the course of IV antibiotics proves to be costly and difficult for patients to tolerate, as they are sequestered away from home, may become bored in the hospital, and may have negative interactions with staff.

Despite provider support for OPAT, hospital policy may frequently prohibit this, which may be due to a lack of institutional inertia/buy-in necessary for implementing interdisciplinary changes on this issue across departments. Extending resources to assist with these unique patient needs has been limited by fears that they may “attract” more volume of this population and that such services are non-reimbursable or create sunk costs. Prior data suggest that high and rising healthcare costs are associated with SBI among PWID, partially driven by longer lengths of stay and higher readmission rates compared to non-PWID [39]. These very real economic burdens can create an environment where focus on short-term cost mitigation outweighs long-term quality improvements. This is likely compounded by multiple competing or misaligned incentives among patients, care providers, and hospital administrators.

Interview findings underscore providers’ interest in strengthening their clinical knowledge/skills through educational resources, consistent with the current literature, which may highlight insufficient addiction medicine education in graduate medical education curricula [28]. McNeely’s work, discussed above, focused on enhancing trainees’ and clinicians’ ability to identify SUDs in medical settings. In our study, participants suggested that even brief interventions, such as attending process groups with patients or shadowing their day, could help provide increased comfort and confidence when working with this population. These low barriers point to feasible changes in standardized education in the inpatient setting. In addition to education, providers emphasized the effectiveness of motivational interviewing in increasing patient engagement.

While recent research has similarly found that most clinicians agree that addiction consult models are urgently needed for OUD, other research recognizes that a one-size approach may not be successful. As described by Evans et al. (2024), the implementation of addiction consults should be responsive to local infrastructure, which may need to address regional and cultural needs [39]. Their study, which interviewed clinical stakeholders across three different geographic areas (i.e., CA, MA, NM), found that economic stability and linguistic and cultural patient-clinician concordance influenced follow-up care.

## Study limitations and future directions

Our study is limited by its single-institution nature and the common limitations of qualitative work, which may affect generalizability, including potential selection bias and the inability to capture every perspective within the diverse provider population that cares for these patients. We attempted to mitigate this by recruiting some participants from external institutions. Participants from the same institution were interviewed, and this data were analyzed by research staff outside the participants’ clinical department with no prior working relationship. However, this could still pose a potential bias. Further, because outpatient locations treating PWID were sparse, we found difficulty in recruiting outpatient providers, particularly in primary care or addiction medicine, who had perspectives on managing SBIs after discharge. Despite this, our findings are essential for hospital-based providers and leaders to consider in enhancing the quality of care for these patients, and indeed emphasize the challenges with linkage to outpatient care. Future research should also incorporate the voices of patients to understand their experiences with hospitalization, discharge, and engagement with outpatient care. Studies assessing outcomes related to the implementation of strategies outlined here will be instrumental in tailoring interventions that are truly helpful in their recovery. It will also be critical for guiding a hospital’s policy and the types of investments it makes as an institution.

## Conclusion

As the field of addiction care strives to become more integrated, there is a growing recognition of the need for multidisciplinary approaches in inpatient care, outpatient follow-up, and care transitions. Our study data highlights the importance of these approaches in the specific context of injection drug-related SBIs and provides concrete strategies that can be leveraged to improve care.

## Supplementary Information

Below is the link to the electronic supplementary material.

Supplementary Material 1

## Data Availability

The data sets analyzed during the present study are available from the corresponding author upon reasonable request.

## Abbreviations

SBI: Severe bacterial infections
SUD: Substance use disorder
PWID: Person who injects drugs
SSTIs: Skin and soft tissue infections
