# Supplementary material for: Improving hospital-based care for patients with injection-drug related infections: provider perspectives
Source: Addict Sci Clin Pract. 2026 Feb 9;21:25. doi: 10.1186/s13722-026-00651-9 (PMC12896020; doi:10.1186/s13722-026-00651-9)
Supplement: Supplementary file 1 — Supplementary Material 1 [file 13722_2026_651_MOESM1_ESM.docx]

**External Stakeholders Interview Guide**

**Introduction:**

*“Hello, my name is __ and I am one of the co-investigators on this study titled “Addressing disparities in treatment of bacterial infections in people who inject drugs” led by the research team at Weill Cornell Medicine. The goal of this interview is to learn about your experiences caring for people who use drugs who are admitted with serious infections. By serious infections, we are referring to bacterial and fungal infections acquired from injecting drugs, like sepsis, endocarditis, osteomyelitis, or skin and soft tissue infections. It is our goal to use the data we collect from providers to design a tailored and responsive intervention for NYP hospitals. We expect this interview will take about 30-60 minutes. After completing the interview, you will be provided with a $50 gift card as a thank you for your participation. Before we start the interview, we will go over the informed consent information and discuss any questions you may have.”*

**General information:**

1. Please introduce yourself and tell me your current job title.
   1. Probe: How long have you been working in your current role?
   2. About what proportion of your time is spent on direct patient care, vs other tasks like administration or research, or education?

**Experience caring for people who inject drugs with severe infection:**
*The next few questions will ask about your role and experiences in caring for people who inject drugs with severe infections.*

1. What is your role in hospital care for these patients?
   1. FOR PHYSICIANS: Do you admit the patients (i.e. primary team)? Do you consult?
   2. FOR NURSING: Which ward do you primarily care for patients (i.e. general floor / med-surg, ER, ICU)?
   3. FOR SOCIAL WORK/CASE MANAGEMENT: Is there a particular ward or clinical department you usually work with?
2. What is your role in outpatient care for these patients?
3. In an average week or month, about how many patients with injection drug use and serious infection do you care for?
4. FOR PHYSICIANS/ NURSE PRACTITIONERS: Please describe your usual approach to arranging antibiotics for patients who require an extended course of antibiotics.
   1. What are the factors that most influence your decision on which patients may go home with antibiotics compared to staying in the hospital or going to a facility?
   2. Are there any other strategies you use for extended courses of antibiotics, such as outpatient infusion centers or long-acting injectable agents?
   3. Probe: Does your approach differ for patients who are currently injecting drugs compared to those with a history of injection drug use? What about patients who are on medications like methadone or buprenorphine?
5. For patients who require substance use treatment, what is your overall approach to providing or referring for substance use treatment services?
   1. What are the factors that influence whether a patient with opioid use disorder receives methadone or buprenorphine in the hospital?
   2. FOR NON-ADDICTION SPECIALISTS: How do you approach referring patients to inpatient addiction specialty consultation? What is that process like?
   3. What about outpatient care after discharge?
6. Patients admitted with infection may request to be discharged before the medical provider thinks they are ready (sometimes called “against medical advice” or “AMA”): Can you describe your approach to patients who request discharge in this fashion?
   1. Probe: How do you arrange for follow-up prescriptions or medical care?

**I’d like to now shift the interview toward discussing interventions or programs in place at your institution to improve care for people who inject drugs with infections. These questions will be focused on 3 areas:**

- **Interventions that have been attempted to be implemented (both successes and failures**
- **Interventions that are ideal for improving healthcare**
- **Interventions that are ideal to improve your ability to care for these patients**

1. To start off: Can you describe any interventions or programs that your hospital offers that are designed to improve healthcare for people who inject drugs hospitalized with infections?
   1. How did the intervention/program get started?
   2. What resources were needed to start the intervention?
   3. What resources, including staffing, are needed to sustain the intervention?
   4. Who were the major champions of clinical leadership? Hospital leadership?
   5. Have the outcomes of the intervention been evaluated? If so, how?
   6. What were the main barriers to the intervention’s success, and how were they overcome?
   7. What are the main features that make this intervention successful?

*Note to interviewer: if they have several programs, ask details about each***

1. Can you describe any proposed interventions that you haven’t implemented that you feel could improve the healthcare of this patient population?

<*Note to interviewer: Use the probes listed in the previous question.>*

1. Are there any strategies that your hospital uses to make that makes it easier for you as a provider to care for these patients? For example, resources or services available to you that you think may not be available at most hospitals?
   1. Probe: How have these strategies been helpful?

How can we motivate physicians to better care for this patient population?

1. Are there any strategies to help motivate physicians and other clinical staff to improve care for this population in the hospital?
   1. Probes:
      1. Community health
      2. Increased trust between patient and provider -> indirectly improved outcomes
      3. Health justice

How can we improve medical education?

1. What are some gaps, if any, do you see in the medical educational curriculum in helping such patients with their hospital care and post-discharge care? This can be at an undergraduate education (medical school), residency, or post-graduate training.

*We are now at the end of the interview. Do you have additional thoughts to share that you would like to add?*

**Conclusion:**

*“We greatly appreciate your time participating in this study. Regarding compensation, we will be issuing your $50 gift card shortly, which should arrive in the next 1-2 weeks.*

**Leadership Interview Guide**

*“Hello, my name is __ and I am one of the co-investigators on this study titled “Addressing disparities in treatment of bacterial infections in people who inject drugs” led by the research team at Weill Cornell Medicine. The goal of this interview is to learn about your experiences caring for people who use drugs who are admitted with serious infections. By serious infections, we are referring to bacterial and fungal infections acquired from injecting drugs, like sepsis, endocarditis, osteomyelitis, or abscesses. We want to use the data we collect from providers to design a tailored and responsive intervention for NYP hospitals. We expect this interview will take about 30-60 minutes. Before we start the interview, we will go over the informed consent information.”*

**General Information:**

1. Please introduce yourself and tell me your current job title.
   1. How long have you been working at your current job?
   2. How long have you been working overall in the field?
   3. About what proportion of your time is spent on direct patient care, vs other tasks like administration or research, or education?

**Experience caring for people who inject drugs with severe infection:**
*The next few questions will ask about your role and experiences in caring for people who inject drugs with severe infections.* <If the respondent provides NO patient care in q1c, can skip to q5>

1. What is your role in hospital care for these patients?
   1. FOR PHYSICIANS: Do you admit the patients (i.e. primary team)? Do you consult?
   2. FOR NURSING: Which ward do you primarily care for patients (i.e. general floor / med-surg, ER, ICU)?
   3. FOR SOCIAL WORK/CASE MANAGEMENT: Is there a particular ward or clinical department you usually work with?
2. What is your role in outpatient care for these patients?
3. In an average week or month, about how many patients with injection drug use and serious infection do you care for?
   1. In the last five years would you say that number has increased, decreased, or stayed about the same?
      1. IF NOT ‘ABOUT THE SAME’: Why do you think that is the case?
4. FOR PHYSICIANS/ NURSE PRACTITIONERS: Please describe your usual approach to arranging antibiotics for patients who require an extended course of antibiotics.
   1. What are the factors that most influence your decision on which patients may go home with IV antibiotics compared to staying in the hospital?
   2. How do you decide which patients might go to a skilled nursing facility?
   3. Do you ever recommend that some patients go to IV infusion centers or return to the ED to receive their IV infusions?
      1. Are there any other considerations you have for providing extended courses of antibiotics?
   4. Probe: Does your approach differ for patients who are currently injecting drugs compared to those with a history of injection drug use? What about patients who are on medications like methadone or buprenorphine?
5. For patients who require substance use treatment, what is your overall approach to providing or referring for substance use treatment services?
   1. How do you typically manage opioid or other drug withdrawals in your hospitalized patients?
      1. Probe: Do you follow any specific guideline documents?
   2. What are the factors that influence whether a patient with opioid use disorder receives methadone or buprenorphine in the hospital?
   3. FOR NON-ADDICTION SPECIALISTS: How do you approach referring patients to inpatient addiction specialty consultation? What is that process like?
   4. Does your approach to providing substance use treatment referrals differ for patients with different types of substance use disorders (e.g. alcohol use disorder, compared to opioid use disorder, compared to meth)?
6. FOR RNs and SWs: What is your approach to assessing and counseling patients about their treatment in the hospital?
   1. Probe: How do you talk to patients about IV antibiotic treatment? Are there any challenges in administering IV antibiotic treatment for this population?
   2. Probe: How do you talk to patients about substance use treatment? Are there any challenges in providing substance use treatment for this population?
   3. Probe: How do you talk to patients about pain management?
7. Patients admitted with infection may request to be discharged before the medical provider thinks they are ready (sometimes called “against medical advice” or “AMA”): Can you describe your approach to patients who request discharge in this fashion?
   1. Probe: What do you think are the main reasons for AMA discharge in this population?
   2. Probe: How do you arrange for follow-up prescriptions or medical care?
   3. Probe: What are the main barriers to arranging follow-up prescriptions or medical care?

**Barriers in receiving healthcare:***We’d now like to ask you a few questions about the barriers that individuals who inject drugs face, compared with patients with similar infections who do not inject drugs.*

1. In your experience, what are the root causes of barriers in receiving healthcare for people who inject drugs while hospitalized with infections? And as we think about this question, I’d like to frame it as barriers potentially originating from the provider end versus barriers originating from the patient end.
   1. Probe: Are most barriers stemming from the healthcare provider or from the patient? What causes these barriers?

**Provider-related barriers**

1. Do you think there is an unwillingness of some providers to offer care interventions?
2. Are providers more likely to deliver medical interventions like procedures and surgery?
3. Do you think some providers have difficulty accessing inpatient treatment or consultation for substance use disorder?
4. Do providers ensure coordinating social work services when indicated/needed?

**Patient-related barriers:**

1. Please describe any instances where you have witnessed any unwillingness of some patients to accept care interventions.
2. How much of an influence do you think stigma and discrimination against persons who inject drugs contribute to patients’ healthcare-seeking behaviors (i.e., willingness to be treated)?
3. Please describe any barriers you believe people who inject drugs face in receiving healthcare after being discharged from the hospital.
   1. Probe: Difficulty accessing follow-up medical care specifically for substance use disorder after discharge?
   2. Probe: Difficulty accessing follow-up medical care, like specialist visits, primary care?
   3. Probe: Difficulty with receiving or taking antibiotics?
4. What differences do you see in the clinical outcomes of patients hospitalized with infections between those who inject drugs versus those who do not inject drugs (i.e. mortality, re-admission rates, infection cure rates)?

**Interventions to improve care for people who inject drugs with infections**

1. Are there any interventions or programs, clinical or educational, that the hospital could offer that would improve healthcare for persons who inject drugs hospitalized with infections?
   1. Probe: Which is the most important one, and tell me about why that would be helpful?
   2. Probe: Which do you think would be the most feasible to implement and why?
2. What types of interventions or programs outside of the hospital, like outpatient or community-based services, do you believe could improve healthcare for people who inject drugs hospitalized with infections?
3. What other types of interventions or programs do you believe would make it easier for you, as a provider, to provide care for this population?
   1. Do you have any experience working on such interventions outside of NYP?

**Financial and organizational considerations:**

1. Can you describe the impact of serious infections among people who inject drugs on your hospital or department’s costs and revenues?
   1. What are the direct or indirect consequences of this?
2. What are the *financial* barriers to implementing new interventions to care for this patient population?
   1. <**Interviewer note:** if participant mentioned specific interventions or programs earlier, please call back to those with this question>
3. What are *organizational* barriers to implementing new interventions? By organizational barriers, we mean things related to organizational structures, workforce management, and other things that act outside an individual clinician’s control.

*We are now at the end of the interview. Do you have additional thoughts to share that you would like to add?*

*Is there anyone else that comes to mind who you think would have helpful perspectives on these topics? (If only physicians suggested: We’re also looking for healthcare professionals other than physicians, like nurses, social workers, etc. Does anyone in those roles come to mind?)*

**Conclusion:**

*“We greatly appreciate your time participating in this study. Regarding compensation, we will be issuing your $50 gift card shortly, which should arrive in the next 1-2 weeks.*

**Provider Interview Guide**

**Introduction:**

*“Hello, my name is __ and I am one of the co-investigators on this study titled “Addressing disparities in treatment of bacterial infections in people who inject drugs” led by the research team at Weill Cornell Medicine. The goal of this interview is to learn about your experiences caring for people who use drugs who are admitted with serious infections. By serious infections, we are referring to bacterial and fungal infections acquired from injecting drugs, like sepsis, endocarditis, osteomyelitis, or skin and soft tissue infections. It is our goal to use the data we collect from providers to design a tailored and responsive intervention for NYP hospitals. We expect this interview will take about 30-60 minutes. After completing the interview, you will be provided with a $50 gift card as a thank you for your participation. Before we start the interview, we will go over the informed consent information and discuss any questions you may have.”*

**General information:**

1. Please introduce yourself and tell me your current job title.
   1. Probe: How long have you been working in your current role?
   2. About what proportion of your time is spent on direct patient care, vs other tasks like administration or research, or education?

**Experience caring for people who inject drugs with severe infection:**
*The next few questions will ask about your role and experiences in caring for people who inject drugs with severe infections.*

1. What is your role in hospital care for these patients?
   1. FOR PHYSICIANS: Do you admit the patients (i.e. primary team)? Do you consult?
   2. FOR NURSING: Which ward do you primarily care for patients (i.e. general floor / med-surg, ER, ICU)?
   3. FOR SOCIAL WORK/CASE MANAGEMENT: Is there a particular ward or clinical department you usually work with?
2. What is your role in outpatient care for these patients?
3. In an average week or month, about how many patients with injection drug use and serious infection do you care for?
   1. In the last five years, would you say that number has increased, decreased, or stayed about the same?
      1. IF NOT ‘ABOUT THE SAME’: Why do you think that is the case?
4. How many years of experience do you have providing care to people who inject drugs who are admitted with infections (training years may also count)?
5. FOR PHYSICIANS/ NURSE PRACTITIONERS: Please describe your usual approach to arranging antibiotics for patients who require an extended course of antibiotics.
   1. What are the factors that most influence your decision on which patients may go home with IV antibiotics compared to staying in the hospital?
   2. How do you decide which patients might go to a skilled nursing facility?
   3. Do you ever recommend that some patients go to IV infusion centers or return to the ED to receive their IV infusions?
      1. Are there any other considerations you have for providing extended courses of antibiotics?
   4. Probe: Does your approach differ for patients who are currently injecting drugs compared to those with a history of injection drug use? What about patients who are on medications like methadone or buprenorphine?
6. For patients who require substance use treatment, what is your overall approach to providing or referring for substance use treatment services?
   1. How do you typically manage opioid or other drug withdrawals in your hospitalized patients?
      1. Probe: Do you follow any specific guideline documents?
   2. What are the factors that influence whether a patient with opioid use disorder receives methadone or buprenorphine in the hospital?
   3. FOR NON-ADDICTION SPECIALISTS: How do you approach referring patients to inpatient addiction specialty consultation? What is that process like?
   4. Does your approach to providing substance use treatment referrals differ for patients with different types of substance use disorders (e.g. alcohol use disorder, compared to opioid use disorder, compared to meth)?
7. FOR RNs and SWs: What is your approach to assessing and counseling patients about their treatment in the hospital?
   1. Probe: How do you talk to patients about IV antibiotic treatment? Are there any challenges in administering IV antibiotic treatment for this population?
   2. Probe: How do you talk to patients about substance use treatment? Are there any challenges in providing substance use treatment for this population?
   3. Probe: How do you talk to patients about pain management?
8. Patients admitted with infection may request to be discharged before the medical provider thinks they are ready (sometimes called “against medical advice” or “AMA”): Can you describe your approach to patients who request discharge in this fashion?
   1. Probe: What do you think are the main reasons for AMA discharge in this population?
   2. Probe: How do you arrange for follow-up prescriptions or medical care?
   3. Probe: What are the main barriers to arranging follow-up prescriptions or medical care?

**Barriers in receiving healthcare:**

*We’d now like to ask you a few questions about the barriers that individuals who inject drugs face, compared with patients with similar infections who do not inject drugs.*

1. In your experience, what are the root causes of barriers in receiving healthcare for people who inject drugs while hospitalized with infections? And as we think about this question, I’d like to frame it as barriers potentially originating from the provider end versus barriers originating from the patient end.
   1. Probe: Are most barriers stemming from the healthcare provider or from the patient? What causes these barriers?

**Provider-related barriers**

1. Do you think there is an unwillingness of some providers to offer care interventions?
2. Are providers more likely to deliver medical interventions like procedures and surgery?
3. Do you think some providers have difficulty accessing inpatient treatment or consultation for substance use disorder?
4. Do providers ensure coordinating social work services when indicated/needed?

**Patient-related barriers:**

1. Please describe any instances where you have witnessed any unwillingness of some patients to accept care interventions.
2. How much of an influence do you think stigma and discrimination against persons who inject drugs contribute to patients’ healthcare-seeking behaviors (i.e., willingness to be treated)?

**Caring for persons who inject drugs versus persons who do not:**

*We’d like to now ask a bit about the differences in caring for this population, compared to patients with similar infections who might not inject drugs.*

1. Please describe any barriers you believe people who inject drugs face in receiving healthcare after being discharged from the hospital.
   1. Probe: Difficulty accessing follow-up medical care specifically for substance use disorder after discharge?
   2. Probe: Difficulty accessing follow-up medical care, like specialist visits, primary care?
   3. Probe: Difficulty with receiving or taking antibiotics?
2. What differences do you see in the clinical outcomes of patients hospitalized with infections between those who inject drugs versus those who do not inject drugs (i.e., mortality, re-admission rates, infection cure rates)?

**Interventions to improve care for people who inject drugs with infections:**

1. Are there any interventions or programs, clinical or educational, that the hospital could offer that would improve healthcare for persons who inject drugs hospitalized with infections?
   1. Probe: Which is the most important one and tell me about why that would be helpful?
   2. Probe: Which do you think would be the most feasible to implement and why?
2. What types of interventions or programs outside of the hospital, like outpatient or community-based services, do you believe could improve healthcare for people who inject drugs hospitalized with infections?
3. What other types of interventions or programs do you believe would make it easier for you, as a provider, to provide care for this population?
   1. Do you have any experience working on such interventions outside of NYP?

*We are now at the end of the interview. Do you have additional thoughts to share that you would like to add?*

*Is there anyone else that comes to mind who you think would have helpful perspectives on these topics? (if only physicians suggested: We’re also looking for healthcare professionals other than physicians, like nurses, social workers, etc. Does anyone in those roles come to mind?)*

**Conclusion:**

*“We greatly appreciate your time participating in this study. Regarding compensation, we will be issuing your $50 gift card shortly, which should arrive in the next 1-2 weeks.* ***What is the best email to send your gift card to?***
